# Supplementary material for: Timing and pacing of the Late Devonian mass extinction event regulated by eccentricity and obliquity
Source: Nat Commun. 2017 Dec 22;8:2268. doi: 10.1038/s41467-017-02407-1 (PMC5741662; doi:10.1038/s41467-017-02407-1)
Supplement: Supplementary file 1 — Supplementary information [file 41467_2017_2407_MOESM1_ESM.pdf]

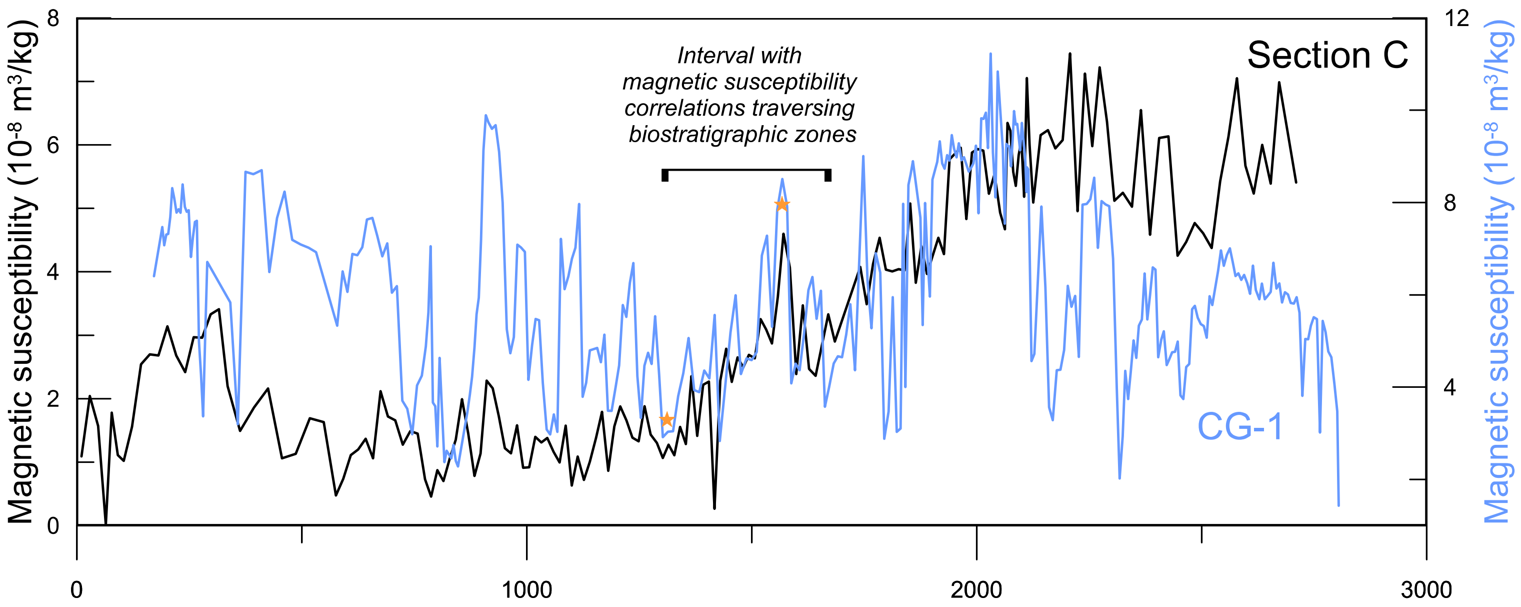

Supplementary Figure 1: Comparison of CG-1 (Iowa) and Section C (western Canada) magnetic susceptibility signals in the time domain. Two correlating lines (here indicated by orange stars) between both sections traverse the biostratigraphic zonation scheme (Zone 11 in CG-1 vs. Zone 12 in Section C). It is unclear whether this discrepancy is caused by diachronism between Iowa and western Canada, an inaccurate biostatigraphic zonation, or erroneous correlation. Yet, the excellent match between the susceptibility signals of these two signals in this interval strongly substantiates our preferred correlating lines (Fig. 5).

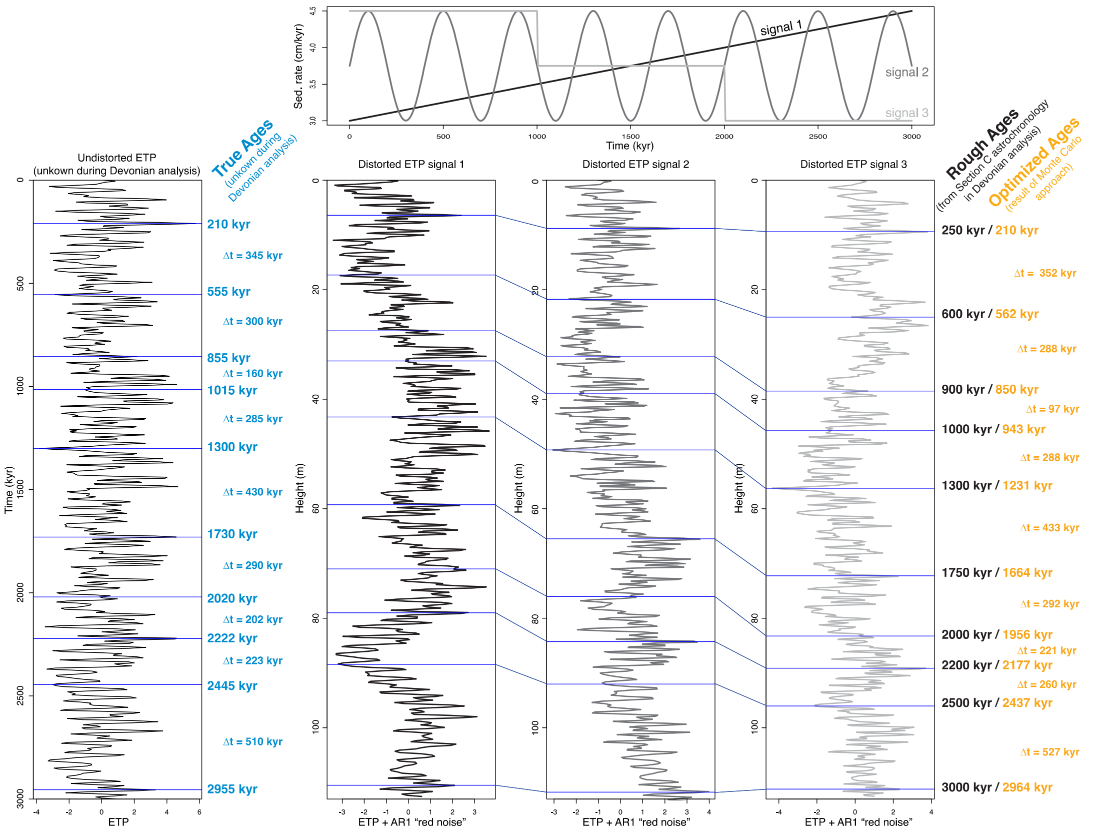

Supplementary Figure 2: Three distorted eccentricity-tilt-precession (ETP) models illustrate the age modelling approach adopted in this study. The three distorted ETP signals were distorted by three different sedimentation rate models (top panel), as well as by adding AR1 “red noise” ($\rho = 1$) of which the variance is equal to the variance of the ETP model. Blue connectors depict ten correlating lines between the three signals, equivalent to the correlations made between the six Late Devonian sections in Figures 5 and 6. Each correlating line is assigned a “rough” relative age based on the interpretation and counting of astronomical cycles in-between correlating lines. In the Devonian analysis, rough ages come from the previously published astrochronology for Section C[1, 2]. In this study, we introduce a Monte Carlo approach that slightly alters the time-differences between correlating lines. After each iteration, we evaluate the astronomical misfit of that particular Monte Carlo simulation. The “optimized” ages, indicated on the figure, represent the best astronomical fit obtained by the Monte Carlo approach (see Figure S4). In this example, one can compare the “optimized” ages to the “true” ages, as the distortion of the original ETP model is exactly known. Though, in the Devonian analysis, the distortion between the forcing and the sedimentary record is not known.

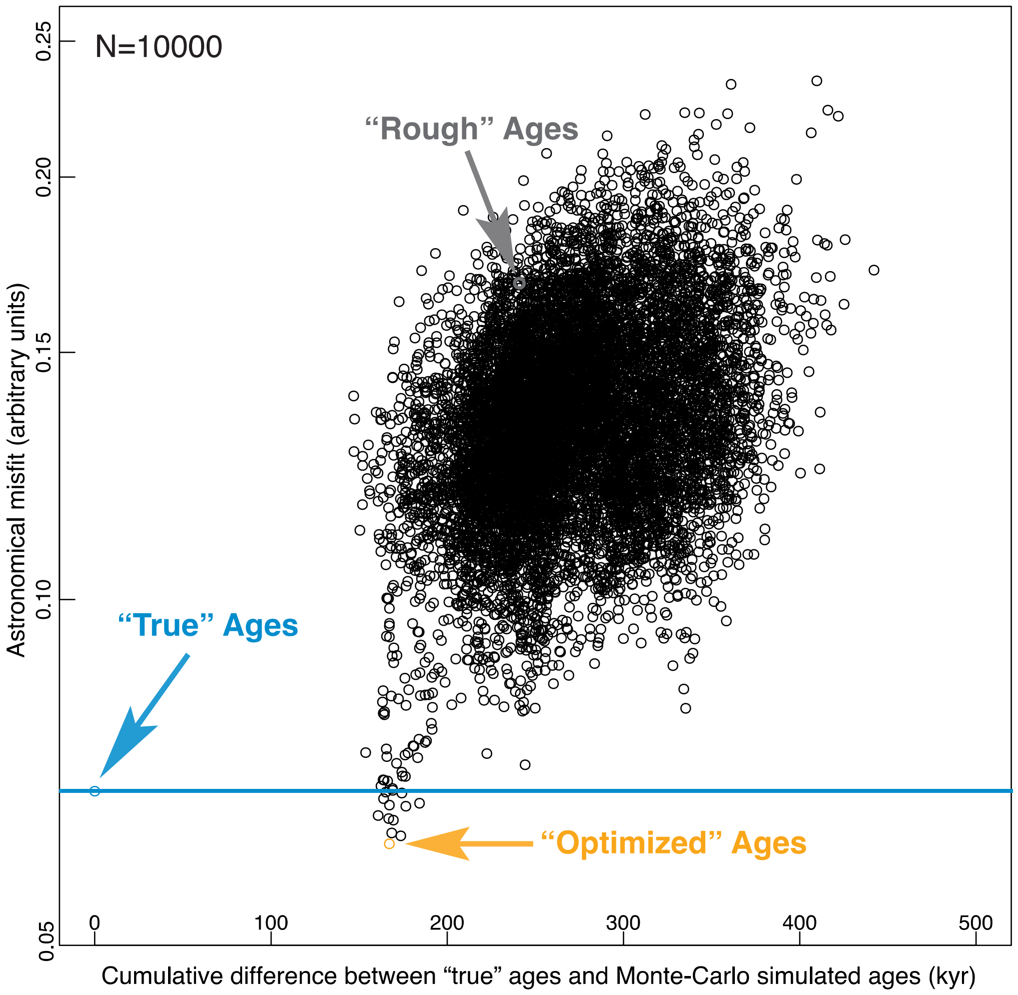

Supplementary Figure 3: The Monte Carlo approach adopted in this study alters the time-differences between correlating lines, and calculates the astronomical misfit of that particular Monte Carlo simulation with the expected orbital frequencies. In this example, the astronomical misfit calculated when using the "true" ages of the correlating line amounts 0.073 (blue horizontal line). This misfit is not zero, as distortion by varying sedimentation rates in-between correlating lines cannot be undone by this approach. The Monte Carlo simulation with the lowest astronomical misfit is retained and labelled "optimized" ages. These "optimized" ages are also shown on Figure S3. The cumulative difference in time-differences between the "true" and "optimized" ages amounts 167 kyr. For the "rough" ages, the cumulative difference with the "true was 240 kyr. The Monte Carlo approach adopted in this illustration thus resulted in an "optimized" age model that is 30% closer to the "true" ages, compared to the "rough" age model. In the Devonian analysis, the "true" ages are obviously not known, which is why we use "optimized" ages as the best estimate of the time-differences between consecutive tie-points.

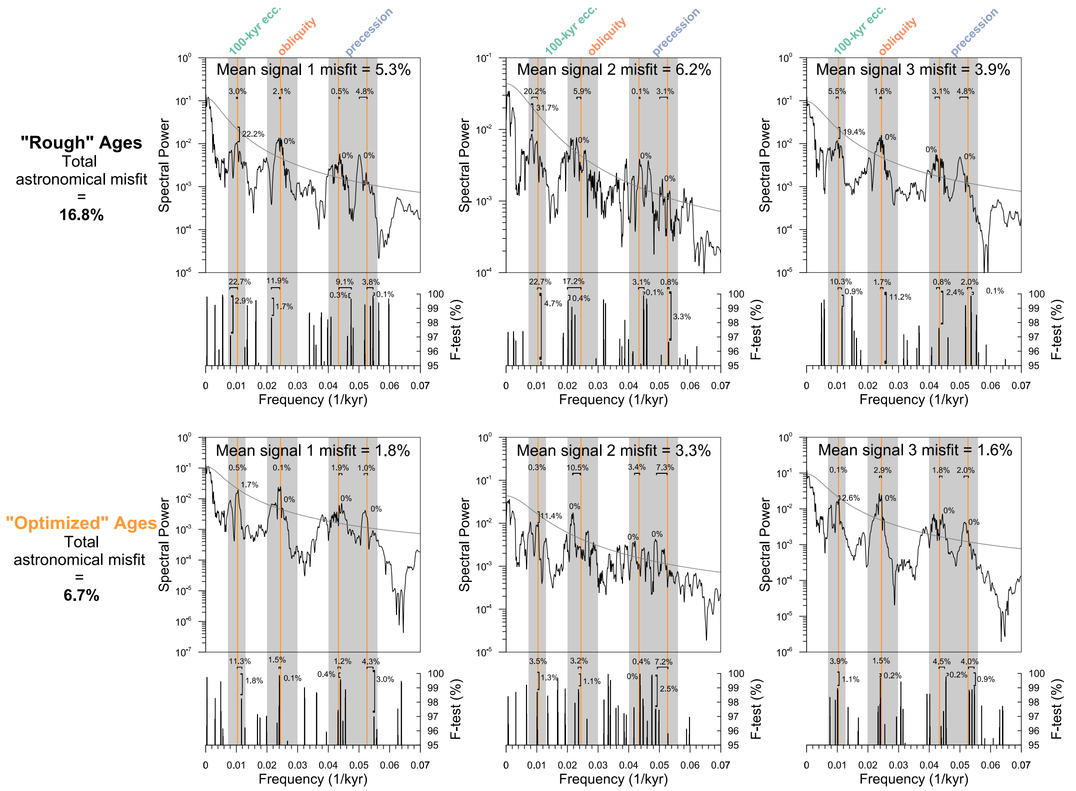

Supplementary Figure 4: Comparison of the power spectra and F-test results of Signals 1, 2 and 3, using the “rough” ages (upper panels) and the “optimized” ages (lower panels). Our age modelling strategy seeks tie-point ages for which the frequencies of spectral and F-test peaks match expected frequencies best, while also favouring high-confidence-level peaks in the different astronomical bands. Here, the “optimized” ages bring a considerable improvement regarding those two characteristics. Orange vertical lines across power spectra indicate the expected astronomical frequencies adopted in the algorithm.

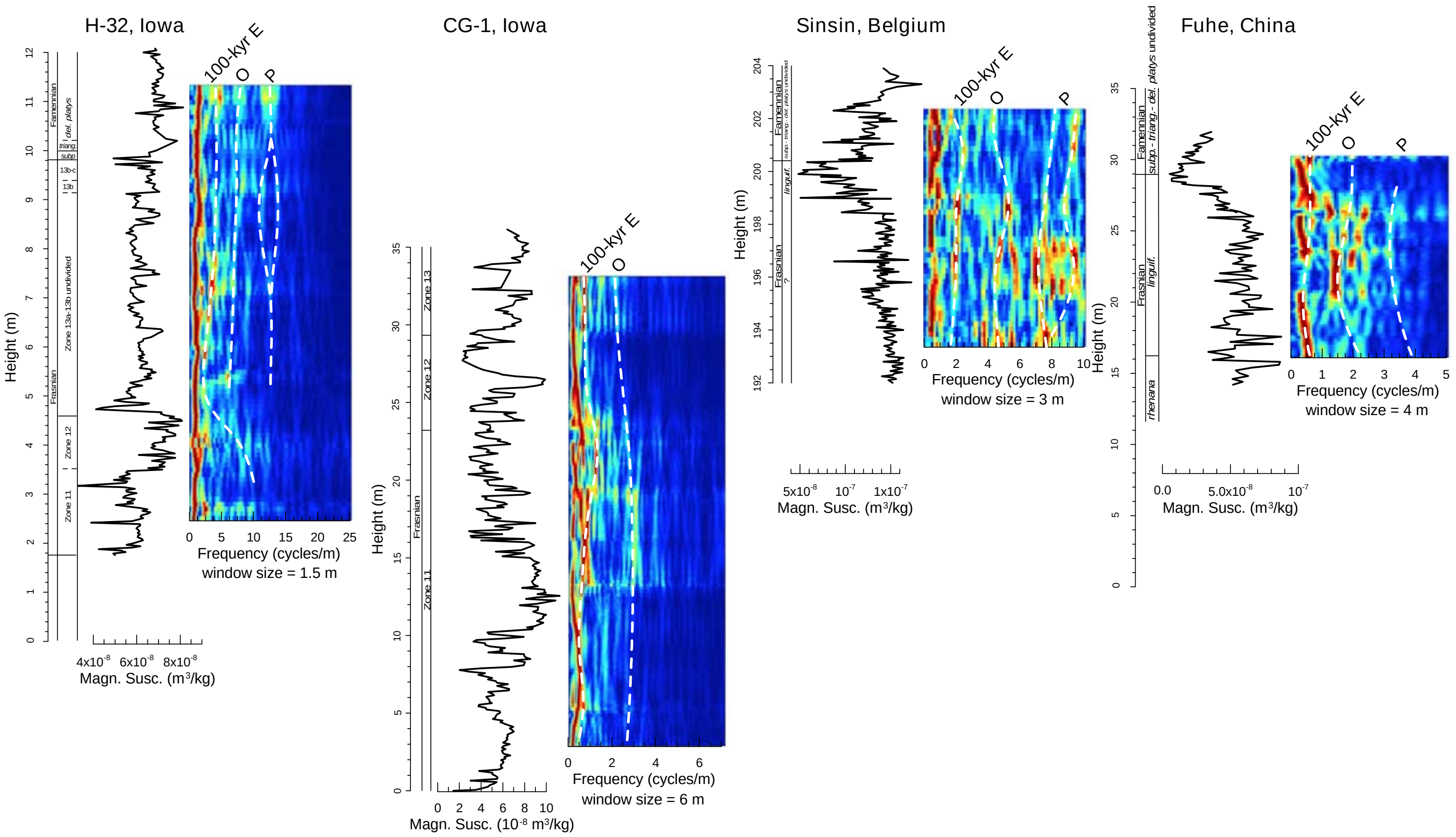

Supplementary Figure 5: Evolutive Harmonic Analyses (EHA) of H-32, CG-1, Sinsin and Fuhe magnetic susceptibility series in the depth domain. The different astronomical components can be traced in the depth domain, demonstrating that these have not been introduced by the age modelling strategy adopted in this paper. 100-kyr E = 100-kyr eccentricity; O = obliquity; P = precession; Magn. Susc. = Magnetic susceptibility.

Supplementary References

1. De Vleeschouwer D, Whalen MT, Day JE, Claeys P. Cyclostratigraphic calibration of the Frasnian (Late Devonian) time scale (western Alberta, Canada). *Geol Soc Am Bull* 2012, **124**(5-6)**:** 928-942.

2. Whalen MT, De Vleeschouwer D, Payne JH, Day JE, Over J, Claeys P. Pattern and Timing of the Late Devonian Biotic Crisis in Western Canada: Insights from Carbon Isotopes and Astronomical Calibration of Magnetic Susceptibility Data. In: Playton T, Kerans C, Weissenberger J, Montgomery P (eds). *New Advances in Devonian Carbonates: Outcrop Analogs, Reservoirs, and Chronostratigraphy*, vol. 107. SEPM: Tulsa, OK, USA, 2017.
